# Practical Notes on Diagnosis and Treatment

**Published:** 1907-08-31

**Authors:** 


### Practical Notes on Diagnosis and Treatment.

**Nose Worry.**

People nowadays of all classes, rich and poor alike, think and worry more over their noses than they did twenty years ago.—*Mr. Stephen Paget.*

**Chorea.**

In severe cases of chorea there is often much wasting, and liberal feeding is an essential part of the treatment. In many cases, too, the free use of brandy is advisable.

**Iodine as a Test for Bile.**

A dilute tincture of iodine floated on the surface of the urine is a convenient test for bile pigment. A positive result is indicated by a green ring at the line of contact.

**Icterus in Enteric Fever.**

Jaundice is a rare complication of enteric fever. When observed it has usually been in the later stages of the disease. It increases the gravity of the prognosis.

**Uranium Nitrate in Glycosuria.**

Dr. C. H. Bond supports Dr. West's conclusions as to the value of uranium nitrate in glycosuria. He prescribes the drug in doses of three grains gradually increased. The dose is given twice daily after food, and never in less than an ounce of water.

**Otitis from Influenza.**

In some of the influenza epidemics otitis has been a frequent complication. In most instances there has been nothing more than a catarrhal inflammation of the middle ear, but in others suppuration has ensued. Severe otalgia and even nerve deafness have also been observed.

**Diabetic Coma.**

Professor Thos. Oliver has recorded a case of this condition successfully treated by saline transfusion. Two and a-half pints of saline fluid (1 dram of chloride of sodium to a pint of boiled distilled water) at 112° F. were introduced into the median basilic vein. The patient regained consciousness during the operation, and four weeks later there had been no relapse.

**Treatment in Perforation of the Stomach.**

The *immediate* treatment is to keep the patient in the horizontal position, and to give opium to relieve the pain and brandy to combat the collapse. But the opium should be used in the form of morphine and by hypodermic injection, and the brandy should be given per rectum, because we need particularly to avoid putting anything into the stomach lest the amount of extravasation into the peritoneal cavity be increased. It is essential to keep the stomach empty, whether you are going to operate or not.—*Mr. Gilbert Barling.*

**Lumbago.**

The application of an ice-bag will sometimes cut short an attack of lumbago.

**Enlarged Glands.**

An ointment of biniodide of mercury, with iodide of sodium, in vaseline, acts well in reducing chronically enlarged glands or an enlarged thyroid.

**Oxygen in Morphine Poisoning.**

In cases of morphine poisoning where there is failure of respiration and marked cyanosis the inhalation of oxygen in association with atropine and strychnine, used hypodermically, has on several occasions proved of distinct service.

**Addison's Disease.**

Though the results are neither so certain nor so impressive as in the case of thyroid extract in myxœdema, there is good reason to prescribe supra-renal extract in Addison's disease. In a certain number of instances decided benefit has followed this practice.

**Puerperal Sepsis.**

In addition to any local measures that may be necessary Dr. Mundé recommends the use of anti-streptococcic serum. He found it successful in three desperate cases where other treatment had failed. From three to six injections were given at intervals of from four to twelve hours.

**Creosote in Tuberculosis.**

In pulmonary tuberculosis creosote is valuable, but it is not a specific. The purest beech creosote should be ordered, and should be given in capsules after food. The dose may be increased to 10 or 15 minims thrice daily. In some cases guaiacol is more readily borne by the stomach.

**Mediastinal Tumour.**

Laryngeal symptoms may be the first evidence of mediastinal tumour—aneurysmal or otherwise. The cough is characteristic. Its chief features are hoarseness and imperfection. Sir Wm. Gairdner long ago taught the importance of "imperfect" cough; he was familiar with its meaning many years prior to the introduction of the laryngoscope.

**Splints in Colles's Fracture.**

Mr. Jonathan Hutchinson says nineteen-twentieths of the cases will do well, and stiffening of the wrist and fingers will be avoided, if the wrist is merely placed between two cushion pads for a fortnight. Only when there is displacement of the carpal fragment which can be removed by extension, and which returns when extension is remitted, should splints be applied. If the routine use of splints were abandoned it would be greatly to the advantage of patients.

